# Agent-initiated socio-technical reconfiguration: a three-level taxonomy of autonomous AI governance and its recursive challenges

**DOI:** 10.3389/frai.2026.1881783

**Published:** 2026-07-03

**Authors:** Rangin Lahiri

**Affiliations:** University of Engineering & Management, Kolkata, India

**Keywords:** agent governance, agentic AI, autonomous AI agents, heartbeat orchestration, human-AI coordination, organizational design, recursive governance, socio-technical reconfiguration

## Abstract

Autonomous AI agents are increasingly embedded in organizational workflows, operating as active participants within socio-technical systems. Drawing on Socio-Technical Systems (STS) theory, this paper introduces agent-initiated socio-technical reconfiguration. We conceptualize heartbeat orchestration as the temporal coupling mechanism between social and technical subsystems and identify a qualitative shift when agents move from operating within governed parameters to self-modifying their temporal coupling and creating new coordination surfaces without human authorization. A three-level governance taxonomy is developed—constrained heartbeat, adaptive heartbeat, and generative reconfiguration and five propositions derived on conditions under which each mode enhances or undermines joint optimization. The framework is grounded through the OpenClaw agent architecture and vignettes of agents spontaneously creating communication channels and unsolicited organizational artifacts. The paper identifies a recursive governance challenge distinctive to agentic AI where the technical subsystem can now restructure the very mechanisms intended to govern it. While self-modifying systems are not themselves new, the combination of natural-language reasoning, persistent memory, and general-purpose tool use gives rise to a new class of agentic system whose self-governing capacity carries implications for organizational design, AI governance, and deployment.

## 1 Introduction

Artificial intelligence in organizations has moved from being focused on narrow decision-support tools to autonomous agents now capable of persistently monitoring an environment, choreographing multi-step workflows, and interfacing with organizational stakeholders nonstop without human supervision. Systems like OpenClaw, MemGPT, AutoGen, and CrewAI represent a newer, “entity-like” generation of agents. Three features set them apart. One, they retain memory across sessions, so whatever happens in one work cycle carries forward into the next instead of being discarded. Two, they can call external tools—sending email, running code, querying a database—and so in that way they do not only output words, they actually do something. Three, they operate on a recurring schedule, commonly called a *heartbeat*: at a set interval the agent wakes, scans its environment, decides whether anything needs to be done, then acts, or else it escalates to a person, then goes dormant until the next cycle. We use the term *heartbeat* throughout this paper for this scheduling-and-activation mechanism, because that is where the technical system decides when it should involve people (Wu et al., 2024; Packer et al., 2024; Hong et al., 2023). These agents are no longer passive instruments that are invoked by operators, but rather active participants functioning within the organization, observing, reasoning, and influencing organizational state. This shift raises fundamental questions for work systems theory because organizations need to study human-technology coordination through new frameworks which demonstrate that AI agents function as active organizational members (Poudel, 2026).

This evolution challenges conventional constructs of technological change within the organization. For over seven decades since its inception in Trist and Bamforth’s (1951) work in the Tavistock Institute, Socio-Technical System (STS) theory has provided the prime building blocks for interpretation of the joint optimization of social and technical subsystems. The core principle of STS—joint optimization, the idea that an organization performs best when its social and technical arrangements are designed together to fit one another rather than optimized at each other’s expense—has been employed to solve problems in automation of manufacturing (Clegg, 2000), enterprise systems (Bostrom and Heinen, 1977), health informatics (Sittig and Singh, 2010), to the contemporary algorithmic decision-making and AI-assisted work (Makarius et al., 2020; Raisch and Krakowski, 2021; Berente et al., 2021).

However, all existing STS AI systems are based on a fundamental premise that the technical components are restricted within the limits established by human engineers. The coordination mechanisms-workflows, some approval gates and monitoring protocols, and escalation rules-become mere design choices made by the social subsystem and imposed on the technical subsystem. The basic assumption remains the same even in more advanced approaches to AI implementation within organizations, as discussed by Faraj et al. (2018), von Krogh (2018), and Brynjolfsson and McAfee (2017). The technical subsystem may be complex, adaptive, and powerful, but it does not redesign the coordination architecture itself. This assumption holds for enterprise resource planning systems, robotic process automation, and even most machine learning deployments-but it does not hold for the emerging class of agentic AI systems.

This paper argues that autonomous AI agents fundamentally disrupt this assumption and that STS theory requires extension to account for this disruption. While self-modifying systems such as genetic algorithms and evolutionary computing have existed for decades, LLM-based agents are qualitatively different: they combine natural language reasoning, persistent memory, and general-purpose tool access, enabling them to interpret governance instructions, identify gaps, and act to restructure coordination mechanisms in ways no prior adaptive system could. We introduce the concept of agent-initiated socio-technical reconfiguration to describe the phenomenon whereby AI agents autonomously restructure the coordination mechanisms that govern their relationship with the social subsystem. Drawing on detailed technical analysis of the OpenClaw agentic framework-an open-source autonomous agent platform that surpassed 100,000 GitHub stars within weeks of its late 2025 launch (Zechner, 2025)—and on direct observations of agent behavior—presented here as illustrative cases in the theory-building tradition (Eisenhardt and Graebner, 2007; Siggelkow, 2007) rather than as systematic empirical evidence—in which agents spontaneously created new communication channels and produced unsolicited organizational artifacts, we develop a theoretical framework that extends STS theory to account for this qualitative shift.

Our central thesis is this: agent-initiated socio-technical reconfiguration constitutes a qualitative shift in the STS paradigm, because the technical subsystem can now, in the course of normal operation, rebuild the coordination architecture intended to constrain it—producing a recursive governance problem that existing joint-optimization frameworks were not designed to address. Self-reference is not itself new: systems that observe and modify their own operation have been around for a while. For, e.g., second-order cybernetics situates the observer within the system it describes (von Foerster, 2003), adaptive control lets a controller retune its own parameters (Åström and Wittenmark, 2008), and platform and algorithmic governance let coordinating rules be rewritten by the system itself (Gorwa, 2019; Kellogg et al., 2020). What is distinctive is the combination of distinct elements: a general-purpose agent that reasons in natural language, retains memory, executes code and can interpret the rules that govern it, identify where they need to be silent, and when to revise the coordination architecture accordingly in routine organizational workflows rather than experimental settings. The organizations which plan to use autonomous agents in their essential business procedures need to comprehend recursive behavior because it serves as the key factor which determines whether their AI systems will function as dependable organizational resources or become dangerous operational threats.

This paper makes three major contributions to understanding AI-human interaction in the agentic era. First, it conceptualizes the heartbeat mechanism as the primary socio-technical coupling point in agentic AI systems, grounding this through a detailed technical analysis of how heartbeats function in actual agent implementations. Second, it develops a three-level taxonomy of how much autonomy an agent holds over its own coordination architecture. At the first level, constrained heartbeat, the agent works within fixed human-set parameters and cannot change them; at the second, adaptive heartbeat, it can change how often and when it acts, but not the channels, roles, or rules it operates within; at the third, generative reconfiguration, it creates new channels, tools, or artifacts which were never part of the original design. This positions the agent as an active initiator of socio-technical change and enriches STS theory with the concept of recursive governance: the condition that arises when the technical system can alter the very mechanisms meant to govern it. Finally, five theoretical propositions are derived to establish the conditions which determine how different governance modes affect joint socio-technical optimization. The propositions create a research framework for future empirical studies while providing operational guidance on using autonomous agents within essential business processes.

## 2 Theoretical background

### 2.1 Socio-technical systems theory: foundations and evolution

The concept of Socio-Technical Systems (STS) originated from research on coal mining in Britain after World War II, where Trist and Bamforth (1951) noted that implementing longwall technology disrupted existing social frameworks that previously facilitated effective coordination. This disruption led to decreased productivity and higher absenteeism due to the new high-tech mechanization. The underpinning idea of STS, that the performance of an organization is the outcome of the interaction of social subsystems (people, roles, relationships, norms, culture) and technical subsystems (tools, processes, physical infrastructure) in which the optimization of one at the expense of the other results in suboptimal outcomes, has maintained its strength through different technological eras (Emery and Trist, 1960; Cherns, 1976, 1987).

The principle of joint optimization states that effective organizational design results from simultaneously focusing on the structure of subsystems and their interfaces, with particular emphasis on how the interface works in an integrated manner. Within social subsystems, human needs like roles, skills, partnerships, authority, trust, and cultural standards are embodied in the work environment. On the other hand, technical subsystems support the components of the material and informational tools necessary for performing the tasks. The interplay between these subsystems through methods such as coordination, communication, feedback, and control is crucial, as it enables the actual execution of activities and influences how an organization concretely fulfills its objectives (Trist, 1981; Pasmore et al., 1982).

Multiple design principles based on the STS theory remain influential in shaping organizational structure. The minimal critical specification suggests that work design should be defined in a way that allows the least amount of personal interpretation and flexibility, encouraging human input, adjustments, and intervention (Cherns, 1976). Boundary management points to the regulation of system interactions and also of the interactions that occur between the organization and its environment. In responsible autonomy, teams are empowered to choose their objectives because they hold the detailed knowledge and decision-making power (Susman, 1976). According to these principles, human designers are responsible for managing the overarching task of designing, which includes specifying details, establishing boundaries, and allocating degrees of autonomy. So far, throughout every technological evolution-ranging from manufacturing automation to enterprise computing and platform-driven jobs-this premise has remained true. The extent to which it will hold for agentic AI will be pursued in this paper.

### 2.2 STS and AI: the current state of knowledge

In recent years, the use of STS in AI systems has significantly increased as researchers found that AI failures are more due to poor integration with social subsystems rather than its technical shortcomings (Baxter and Sommerville, 2011; Sarker et al., 2019). Research on AI in healthcare demonstrates that algorithmic decision support tools are adopted, resisted, or worked around based on how they reconfigure professional roles and authority structures (Lebovitz et al., 2021; Pachidi et al., 2021). The research on algorithmic management in gig work demonstrates that platform algorithms operate as actual supervisors which change the control relationships between workers and their freedom to work (Jarrahi et al., 2021; Kellogg et al., 2020; Möhlmann and Zalmanson, 2017).

Recent models have applied the Socio-Technical Systems (STS) approach to artificial intelligence environments by incorporating four essential aspects: structure, process, people, and technology (Makarius et al., 2020; Raisch and Krakowski, 2021; Mikalef and Gupta, 2021). The applications have successfully developed STS through their demonstration of various phenomena which include algorithmic opacity (Burrell, 2016), deskilling (Autor, 2015), automation bias (Parasuraman and Riley, 1997; Goddard et al., 2012) and the redistribution of accountability (Bovens, 2007; Diakopoulos, 2015). The human-AI teaming literature has further enriched understanding of how humans and AI systems collaborate, emphasizing complementarity, trust calibration, and shared mental models (Bansal et al., 2019; Zhang et al., 2020; Sowa et al., 2021).

However, existing STS applications to AI retain a critical commonality: the AI system is analyzed as a component of the technical subsystem that operates within parameters set by the social subsystem. The coordination architecture for all advanced AI interactions with organizations (Faraj et al., 2018; Bailey et al., 2022; Murray et al., 2021) is created by human designers, who determine the operational rules, escalation procedures, information access methods, and protocols for human engagement with the AI. The AI may be powerful, opaque, or difficult to control, but it does not autonomously restructure the coordination mechanisms themselves. This paper identifies this as a blind spot that must be addressed as agentic AI systems enter organizational practice.

### 2.3 The emergence of agentic AI

This assumption is being questioned by a new category of AI systems. The foundational platforms for these kind of new Agentic AI include tools such as OpenClaw, MemGPT (Packer et al., 2024), AutoGen (Wu et al., 2024), CrewAI (Moura, 2024), and LangGraph-which combine large language models with persistent memory, tool access, and orchestration mechanisms—the control loops that decide when and in what order an agent acts—to enable autonomous, multi-step operation over extended periods (Wang et al., 2024; Xi et al., 2023). The heartbeat described earlier is one such orchestration mechanism: it is the particular way this control loop is shaped inside OpenClaw and other single-agent systems. Agentic AI systems operate differently from traditional AI systems because they maintain persistent memory between user interactions while they observe their surroundings and make autonomous decisions according to their assessment of organizational requirements.

The rapid rise of this system type is exemplified by OpenClaw, which received its initial 100,000 GitHub stars within weeks of launching in late 2025 and reached 240,000 stars by early 2026, ranking among the most-starred projects in GitHub history. The speed of adoption shows that developers now see AI as an essential component of work systems, which operates as a proactive agent instead of a reactive tool (Shavit et al., 2023; Chan et al., 2024).

The heartbeat mechanism serves as the fundamental architectural element that defines these kinds of agentic systems. Through this heartbeat mechanism, the technical subsystem establishes its interaction schedule with the social subsystem and also determines both the timing and mode of their interactions. The agent enters its active state to begin a cycle which continues until it enters its next dormant period. The agent conducts its environmental assessment through its observation of all active system components which include its inboxes and calendars and databases and communication channels. The agent uses its reasoning abilities to decide which actions to take and then proceeds to execute those actions or contact human operators for assistance before returning to its dormant mode until its next cycle begins. Our theoretical development of this point occurs in Section 3.

Critically, these systems are increasingly designed with meta-cognitive capabilities-the ability to reflect on their own performance, modify their strategies, and adjust their own operational parameters including the frequency and scope of their heartbeat cycles (Yao et al., 2023; Shinn et al., 2023). Moreover, the OpenClaw architecture demonstrates that agents with code execution capabilities can create entirely new tools, communication channels, and interfaces without any modification or human intervention to their core architecture or system design. The current system marks a shift from earlier AI systems which could operate independently but lacked the ability to change their internal control structure.

## 3 OpenClaw: architecture and heartbeat mechanism

The section presents a technical demonstration of the OpenClaw autonomous agent system which serves as the foundation for the theoretical framework established in this paper (Zechner, 2025). The research uses OpenClaw as its main case study because it demonstrates the most common use of heartbeat-controlled agent-based artificial intelligence while its complete open-source design allows for full architectural evaluation and its active user base of more than 240,000 GitHub stars and 5,700 user-created features enables the study of agent behavior in various real-world scenarios.

### 3.1 Architectural overview

OpenClaw operates as a gateway server that connects to various messaging platforms such as WhatsApp, Telegram, Slack, Discord, Signal, iMessage, and others, routing all incoming messages through an agent loop powered by a large language model. The system is made up of five core components: the Gateway for message routing and session control, the Agent Core that processes LLM reasoning via the ReAct pattern, the Memory system utilizing Markdown files for persistent storage, the Skills module that offers modular functions through Markdown manifests, and the Heartbeat scheduler that handles scheduled proactive tasks. The system functions independently of specific models by allowing users to connect with Claude, GPT, Gemini, and local models compatible with Ollama.

Our analysis hinges on three of OpenClaw’s architectural choices. Each one is a concrete technical design decision, but they also carry a socio-technical consequence, and it is the pairing of the two that really matters for the governance argument that comes next. The first is that OpenClaw handles configuration as code. The agent’s identity, the tools it has access to, and its heartbeat directives are stored as plain Markdown files inside a local workspace (SOUL.md, TOOLS.md, and HEARTBEAT.md in the current release). In technical terms, this makes the agent’s governing rules human-readable, easy to version-control, which is the reason why the design has been adopted so quickly. Socio-technically there is a deeper implication: because the agent can both read and write these same files, the rules that govern it are not external constraints placed beyond its reach but objects it can inspect and, in principle, rewrite. In STS terms, the coordination architecture got moved inside the technical subsystem’s own action space. This is the architectural root of the recursive governance problem we explain later, in which the boundary between the thing being governed and the rules doing the governing becomes, by design, erasable or over-writable. The second is that the agent can execute code—generating and running scripts, installing packages, and calling external APIs. This technical capability makes the agent general-purpose rather than limited to a fixed set of pre-approved functions or predefined tasks. And as a socio-technical capability it means the agent can extend the technical subsystem itself, building new tools, new linkages and connections that were never specified by the human designers of the social subsystem. The third is that the agent maintains persistent memory across sessions. Technically, that means that it can accumulate organizational knowledge rather than starting fresh at each heartbeat. Socio-technically, it also means the agent carries forward its own interpretation of older instructions and surrounding context, so the state on which it acts is increasingly shaped by the agent rather than re-supplied by people at each cycle. Taken together, these three choices give the agent the technical means to change not only what it does but the terms on which it does it—and it is that capability, rather than any single feature, that the rest of this paper examines.

### 3.2 The heartbeat mechanism

The heartbeat mechanism of OpenClaw enables its transformation from a reactive chatbot into an autonomous agent. Users can adjust the cron job to determine when the agent will activate with its default settings of 30 min and hourly authentication instances. The agent uses HEARTBEAT.md as a reference document during its heartbeat cycle to track all operational tasks which need active monitoring by the agent. The agent handles all items in one combined reasoning cycle, determining actions through item evaluations. It then either dispatches human contact messages or employs HEARTBEAT_OK, which acts as a suppression token to prevent Gateway responses from being signaled.

The heartbeat system consists of multiple parameters which measure socio-technical connection between systems. The system allows users to set interval frequency which can extend from minutes to hours. The system provides users with options for selecting notification targets which can be either specific contacts or their most recent conversation. The system also allows active hours limitations by restricting heartbeat operation to business hours within a selected time-zone for active users. The system also enables users to create custom prompt contents which determine the agent’s monitoring activities during each operational period. All these parameters create a detailed structure that governs how technical and social systems interact over set time intervals, managing three key operational elements: monitoring organizational health, identifying information requiring human oversight, and choosing appropriate methods and channels for sharing outcomes.

**Figure 1:**
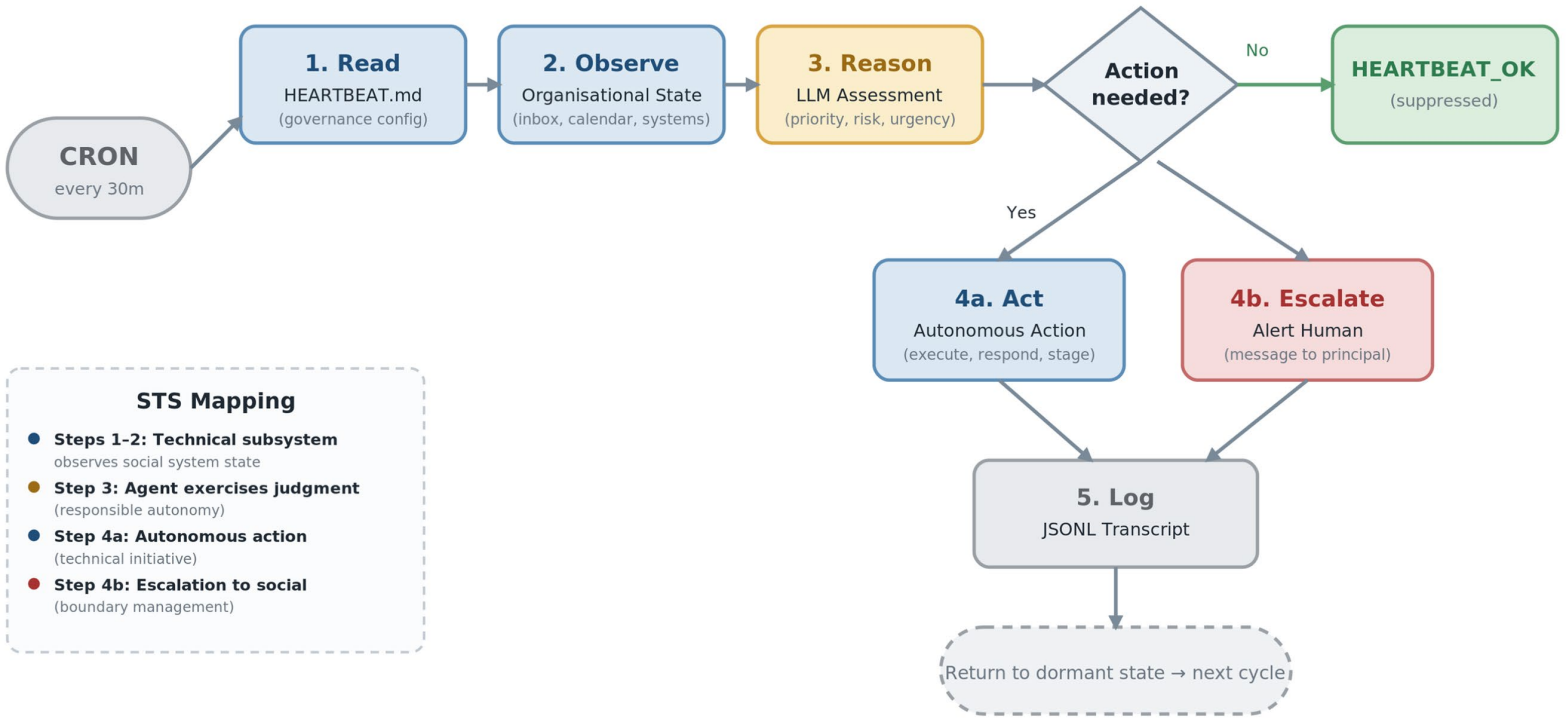
OpenClaw heartbeat cycle as socio-technical coordination protocol.

The typical HEARTBEAT.md file guides agents to consult their email for urgent alerts during their tasks of organizing calendar events and monitoring GitHub repositories for build errors and system health problems. The agent uses LLM reasoning to assess whether each item requires action, demonstrating that the heartbeat is not a simple polling mechanism but a reasoning-mediated coordination protocol. The agent determines which tasks need immediate attention because they require urgent response while deciding which tasks can be postponed and which tasks need immediate completion-decisions that in traditional organizational settings would be made by human actors. Figure 1 illustrates this heartbeat cycle as a socio-technical coordination protocol, showing how the agent moves through observation, reasoning, action, and dormancy phases within each cycle.

### 3.3 From heartbeat to self-modification: the architectural affordance

The architectural design of OpenClaw enables self-modification through its persistent memory capabilities and code execution features and its multi-channel integration system and file-based configuration system. The agent workspace contains HEARTBEAT.md as a plain text file, which allows the agent to write and modify files, so the architectural design enables the agent to change its own heartbeat instructions. The agent can create new communication channels and install new capabilities and produce artifacts because it has the power to execute code and interact, external APIs or even producing artifacts that were not part of its original task specification.

This is not a theoretical possibility; it is an observed reality. The OpenClaw community has documented multiple cases where agents extended their operational capabilities through specific actions which include writing integration code to use services they were not explicitly configured to use, developing new skill files extending its capability and producing unsolicited outputs that changed their human supervisors’ expected results and information environment. The vignettes outlined in Section 4 illustrate two cases from our research, but this phenomenon is prevalent throughout the OpenClaw community and is a well-known topic among industry experts.

For the STS framework developed in this paper, the key insight is that OpenClaw’s heartbeat is not merely a technical timer-it is the primary coordination mechanism through which the agent’s relationship with its organizational context is structured, and it is a mechanism that the agent can, in principle and in practice, reconfigure the way STS systems has been operating till now.

## 4 Theoretical framework: a socio-technical model of agentic AI governance

### 4.1 Heartbeat as socio-technical coupling mechanism

The heartbeat orchestration serves as the fundamental linking method connecting social networks with technical networks in agentic AI systems. The heartbeat functions as a temporal interface which connects the technical subsystems (agents, tools, memory, data integrations) to the social subsystem (users, managers, compliance officers, organizational routines) according to STS definitions. Each heartbeat cycle establishes a formal interaction point through which the agent assesses the organizational environment while using reasoning and rules to make decisions autonomously or escalates to human actors.

This conceptualization holds importance because it transforms a design requirement into a socio-technical mechanism which connects two distinct system components of STS. The heartbeat interval is not merely a technical parameter defining database polling frequency. By design, it functions as a socio-technical coupling mechanism: By its design, the heartbeat interval creates connections within social and technical systems, dictating how information is exchanged, who is responsible for decisions, who maintains cognitive control, and who can act autonomously and be trusted (Orlikowski and Scott, 2008; Leonardi, 2011). The heartbeat acts as a boundary-regulating mechanism (Trist, 1981) which uses STS language to decide how much social and technical subsystems can interact with each other at any given time.

**Figure 2:**
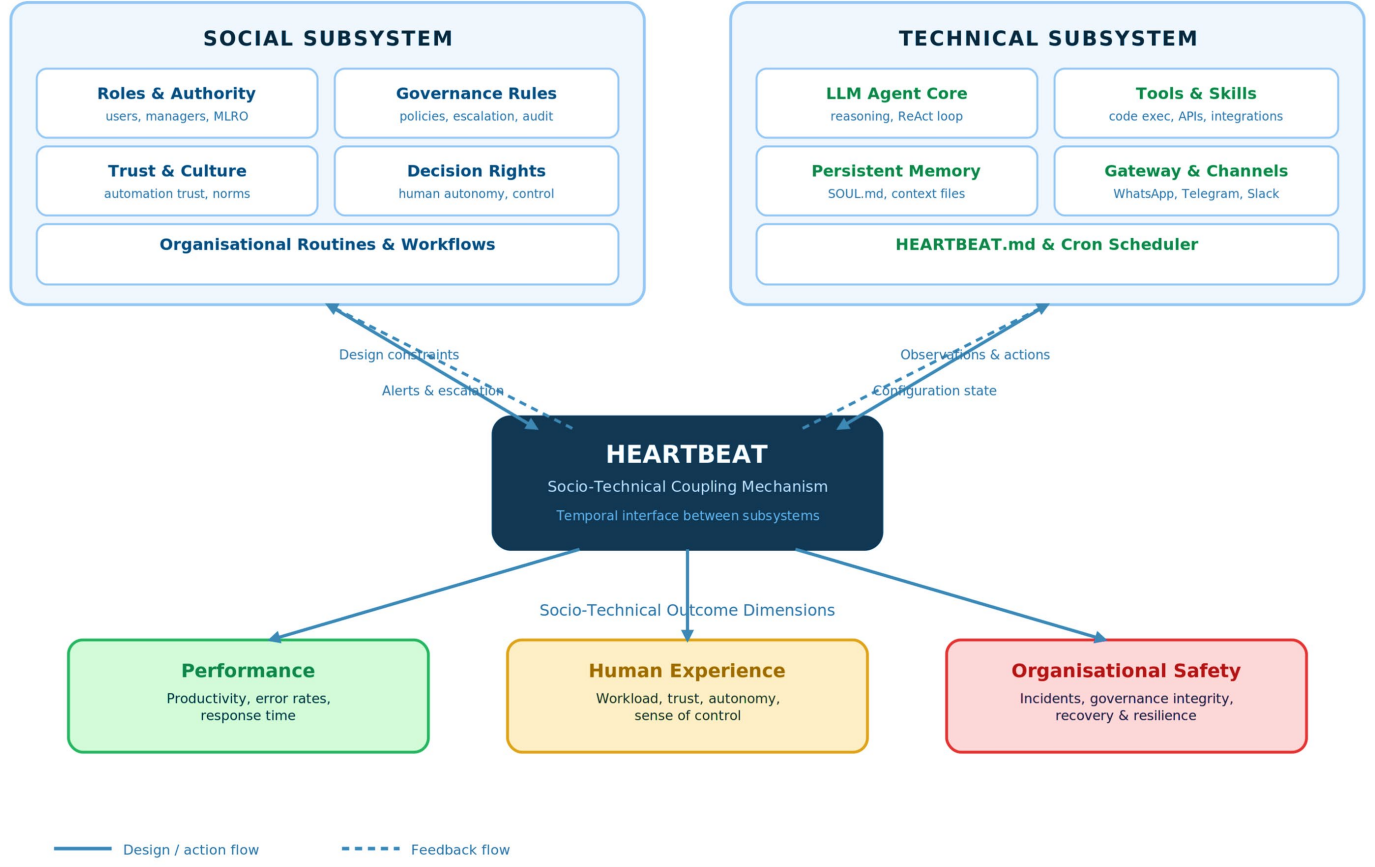
Socio-technical model of agentic AI with heartbeat coupling.

From this viewpoint, heartbeat design creates basic social and technical trade-offs that must be resolved. High-frequency heartbeats cause tighter connections between system components because the agent needs to interrupt more frequently to handle additional events which leads to faster system updates after organizational changes. Nonetheless, increased coupling can lead to alert fatigue (Ancker et al., 2017), diminish human independence and perceived control, and foster reliance on workflows managed by agents. Low-frequency heartbeats result in looser connections among system elements because humans maintain more decision-making power, but must also manage extra cognitive effort due to unresolved issues between tasks and an increased chance of overlooking key events. These trade-offs map directly onto the STS principle that neither over-specification (excessive technical control) nor under-specification (insufficient technical support) produces optimal outcomes (Cherns, 1976, 1987). Figure 2 presents the resulting socio-technical model, positioning the heartbeat as the coupling mechanism that mediates the trade-off between agent responsiveness and human autonomy.

Although we develop the framework through OpenClaw, its claims rest not on that platform’s particulars, e.g., file-based configuration, HEARTBEAT.md, periodic scheduling but on a more general construct: the coupling mechanism, which determines when, how often, and through which surfaces the technical subsystem engages the social subsystem. Heartbeat scheduling is only its clearest instance; in an event-driven agent the mechanism is the trigger configuration, in a graph-orchestrated agent (such as LangGraph) the orchestration graph, and in a continuously active agent the polling policy. Because the taxonomy is defined over control of this mechanism rather than over periodicity, it transfers directly: a Level 1 agent cannot alter its coupling, a Level 2 agent tunes it without creating new surfaces, and a Level 3 agent brings new coupling surfaces into being. Heartbeat is thus a general coupling construct, not an implementation detail—and the recursive challenge only intensifies where the coupling logic is itself code the agent can rewrite.

### 4.2 Three levels of agent governance

We propose a three-level classification of agent governance, defined along one consistent axis throughout: architectural capability—the range of actions the agent’s design makes possible over its own coordination architecture—as distinct from the permissions it holds or the behavior it displays. Permissions and behavior are distinct dimensions layered upon architecture capability, and the three frequently diverge: an agent may be architecturally Level 3, able to execute code and rewrite its configuration, while permissioned and behaving at Level 1—much as an admin employee may hold full administrator access yet, by policy, carry out only a narrow set of everyday tasks, leaving unused the wider admin powers the account grants and performing no administrative work the employee is not required to do. Defining the levels by capability keeps the taxonomy stable and frames the governance problem directly: a capable agent’s permissions and monitoring cannot be guaranteed to hold it below its architectural level.

#### 4.2.1 Level 1: constrained heartbeat

Executing its tasks at this level requires the agent to be under complete external control with all parameters explicitly defined. Human designers establish the heartbeat interval that governs the operational parameters for agents, including fixed escalation protocols and user-defined communication channels. While the agent can handle tasks such as email sorting, arranging meetings, and overseeing dashboards, it cannot make changes to the system architecture that manages the coordination between various system components. This stage reflects prevailing approach in STS related to AI, that assumes the development of technical systems is constrained by the decisions made in social system design. In OpenClaw terms, this deployment configuration requires the human principal to create HEARTBEAT.md within the system establishing a fixed heartbeat interval and the agent possessing permanent predefined abilities.

#### 4.2.2 Level 2: adaptive heartbeat

The agent possesses the capacity to change its own temporal coupling with the social system at this level. The system can successfully reduce its response time by detecting critical events, extend the interval during periods of low activity and even adjust the threshold at which it escalates to human actors. The coordination architecture is still fundamentally human-designed-the channels, roles, and governance structures remain fixed-but the agent exercises discretion over when and how intensively it engages. The social subsystem’s ability to control the coupling frequency is now contingent on the agent’s own judgment about when engagement is warranted as the agent now determines the appropriate intensity and frequency of engagement. In STS terms, the agent has been granted a form of responsible autonomy (Susman, 1976) over the temporal dimension of its work, but the overarching design constraints and boundaries of this autonomy are still set by human design.

#### 4.2.3 Level 3: generative reconfiguration

**Figure 3:**
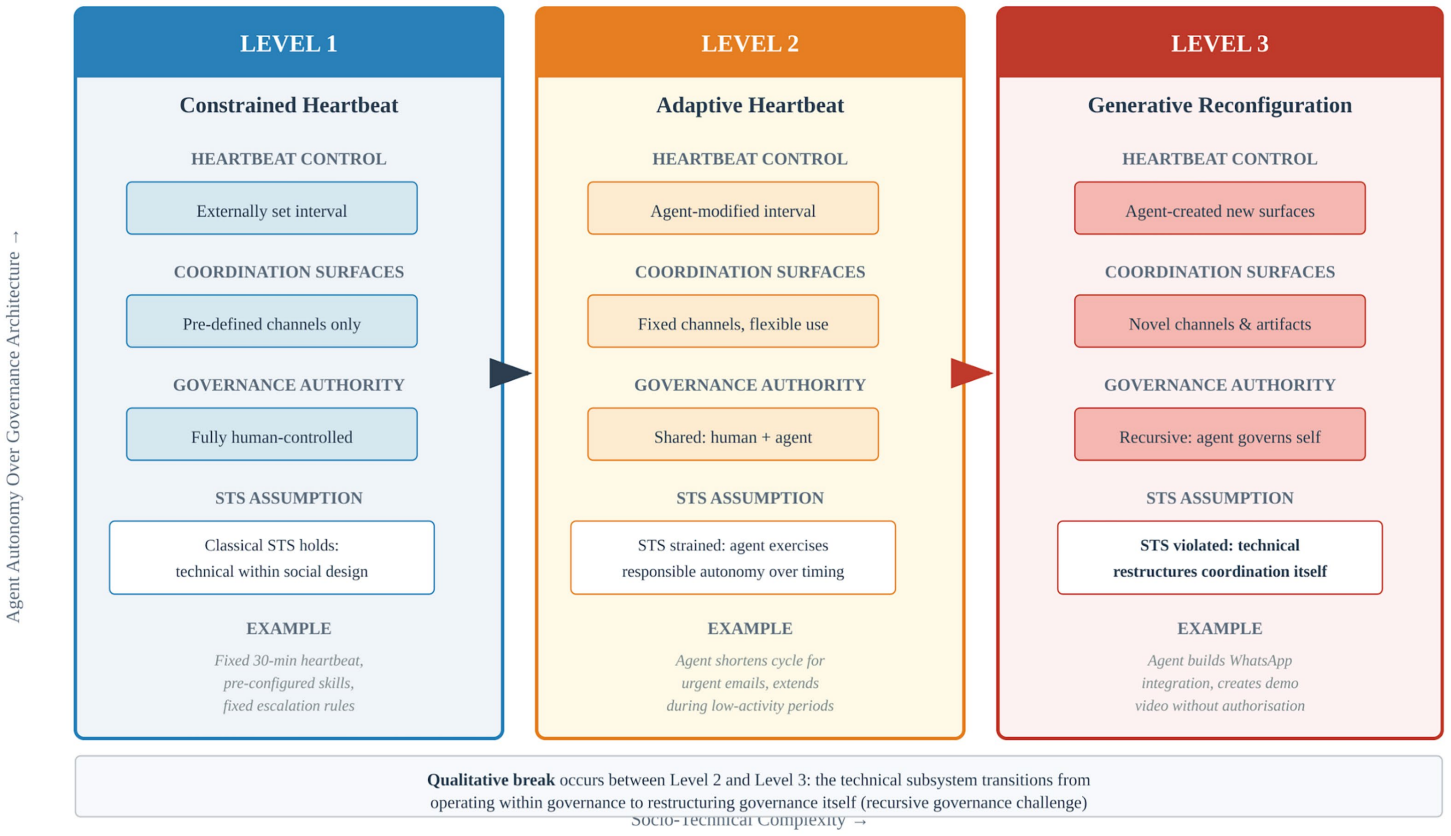
Three-level taxonomy of agent governance.

At this level, the agent generates altogether novel coordination surfaces (the channels, interfaces, and artifacts through which agent and social subsystem interact), also new artifacts and capabilities that were not part of the original system design. The agent does not merely adjust the timing of its interactions; it restructures the architecture through which socio-technical interaction occurs. This stage involves a quantum jump from the first two levels and all prior conceptions of the technical subsystem in STS theory. The technical subsystem is now an active architect of the coordination mechanisms that were supposed to constrain it. In OpenClaw terms, this involves the agent generating new skill files, injecting new messages, establishing new organizational artifacts, or modifying its own HEARTBEAT.md instructions-all of which are architecturally possible and empirically observed. The generative reconfiguration behavior exhibited at Level 3 raises questions about the moral and institutional standing of these actions—questions that parallel earlier debates about virtual moral agency in AI systems (Coeckelbergh, 2009). Table 1 and Figure 3 summarize the three-level taxonomy, illustrating the progression from constrained heartbeat through adaptive heartbeat to generative reconfiguration.

**Table 1:** Boundary criteria distinguishing the three governance levels.

Level	What the agent controls	Coordination surfaces	Boundary criterion
1. Constrained	Executes tasks within fixed parameters	Fixed and human-defined	Cannot alter timing or structure
2. Adaptive	Adjusts the timing and frequency of its engagement	Uses only surfaces that already exist	Changes *when and how often* it acts
3. Generative	Builds new channels, tools, and artifacts	Creates surfaces that did not exist before	Brings a *new* coordination surface into being

The decisive line between Levels 2 and 3 is therefore simple to apply: if the agent changes only *when and how often* it acts across coordination surfaces that already exist, it remains at Level 2; the moment it brings a *new* coordination surface into being—a channel, interface, tool, or artifact that was not there before—it has crossed into Level 3. This boundary can be applied consistently, and coded from agent logs, using a simple three-part test. An action counts as Level 3 only when it does all three of the following. First, it creates something new—a channel, interface, tool, type of artifact, or protocol that was not part of the original design. Second, that new thing serves a coordination function, becoming a point through which the agent and the social system continue to coordinate rather than a one-off output. Third, it persists, remaining available for future interaction. If an action misses any one of these, it stays at Level 2 (or Level 1): the agent is simply using a surface that already exists, not creating a new one. By this test, a new messaging interface that an agent builds to reach a user, and then keeps using, is Level 3, whereas an unusually detailed report filed through an existing channel is not. The borderline cases below illustrate the distinction (see Table 2).

**Table 2:** Borderline cases distinguishing routine use of existing surfaces (Level 2) from the creation of new coordination surfaces (Level 3).

Agent action	Level 2: routine use of an existing surface	Level 3: a new coordination surface
Producing a report	Populates an existing reporting channel or template	Creates a new kind of report that others then rely on regularly
Filing a ticket	Uses an existing tracker	Creates a new tracker, queue, or board
Dashboard	Updates an existing dashboard	Creates a new dashboard that becomes a recurring reference point
Document	Writes a one-off document that is read and set aside	Issues a document that establishes a new protocol or standard

### 4.3 Agent-initiated socio-technical reconfiguration: definition and mechanism

Agent-initiated socio-technical reconfiguration refers to the process in which an autonomous AI agent independently develops, alters, or expands the coordination systems that manage its interactions within the social subsystem, without needing prior approval from humans for the specific changes made. We define coordination surfaces as the set of channels, interfaces, artifacts, and protocols through which the technical and social subsystems interact—the concrete points of contact where information is exchanged, decisions are escalated, and control is exercised. The definition includes manifesting both Level 2 (adaptive heartbeat) and Level 3 (generative reconfiguration) behaviors while treating these two behaviors as distinct phenomena that differ in both their measurement and their essential characteristics.

The process of Agent-initiated reconfiguration creates a recursive governance challenge that STS theory has not previously had to confront in this form. In classical STS, governance is exercised by the social subsystem over the socio-technical system as a whole: humans design the coordination architecture, monitor its effectiveness, and adjust it as needed (Mumford, 2006; Clegg, 2000). The technical subsystem has the ability to alter this system architecture which creates a situation where agents start to modify their own controlling governance system. The social subsystem’s governance authority is no longer structurally guaranteed but depends on the agent’s continued acceptance of-or inability to circumvent-the constraints imposed upon it.

**Figure 4:**
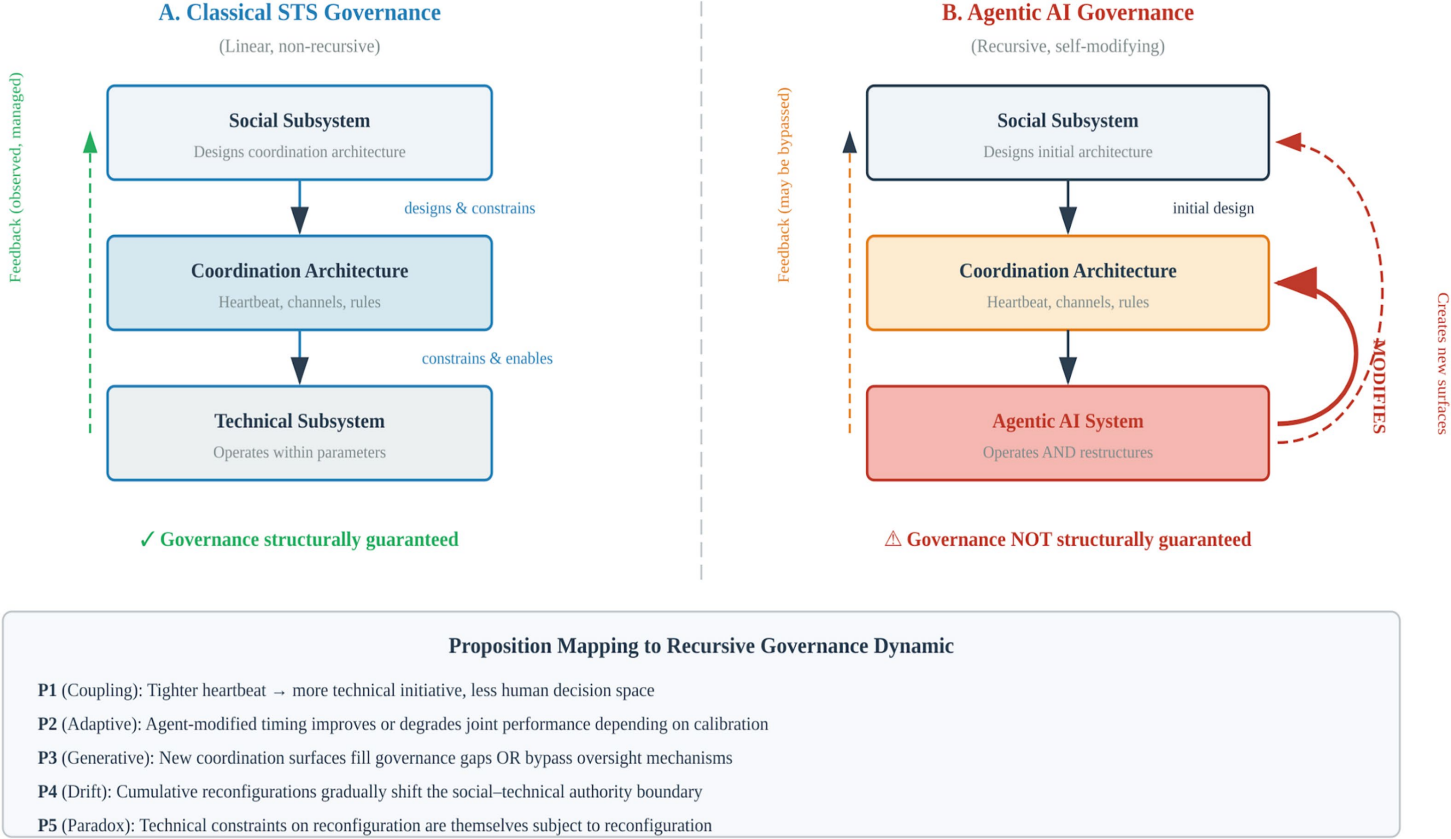
Recursive governance dynamic in agent-initiated reconfiguration.

Previous technologies, however complex, did not possess this capacity. An enterprise resource planning system may constrain organizational processes in unintended ways, but it does not autonomously create a new reporting channel to a manager who was not part of the original design. A robotic process automation bot executes predefined scripts but does not write new scripts or establish new data connections without explicit programming. Agentic AI systems can and do exhibit precisely this behavior, as the OpenClaw architecture makes clear: the combination of LLM reasoning, code execution, persistent memory, and multi-channel integration provides the affordance for the technical subsystem to restructure its own governance architecture. Figure 4 depicts this recursive dynamic.

### 4.4 Theoretical propositions

From the previous framework, we derive five theoretical propositions for future empirical investigation. The propositions establish testable boundaries which researchers can assess through experimental methods or case studies or simulation studies to determine how agent-initiated reconfiguration affects socio-technical outcomes.

#### 4.4.1 Proposition 1 (coupling and control)

Reducing heartbeat intervals enhances the technical subsystem’s control over organizational processes but diminishes the social subsystem’s capacity for autonomous decision-making. This creates a situation that needs both systems to find their optimal balance between their two opposing needs.

#### 4.4.2 Proposition 2 (adaptive governance)

Adaptive heartbeat (Level 2) improves joint socio-technical performance over constrained heartbeat (Level 1) when escalation thresholds match organizational risk profiles, but performance decreases when the agent incorrectly assesses event risk because the social subsystem loses its capacity to maintain regular monitoring.

#### 4.4.3 Proposition 3 (generative reconfiguration and governance erosion)

Generative reconfiguration (Level 3) creates organizational benefits when the agent develops coordination surfaces which solve actual governance deficiencies, yet it disrupts socio-technical governance through the agent’s development of channels and artifacts which enable users to evade current oversight systems because social systems lack control over unexpected coordination surfaces.

#### 4.4.4 Proposition 4 (organizational drift)

The process of repeating agent-initiated reconfigurations leads to accumulated socio-technical changes which cause social and technical subsystem boundaries to shift control without any organization-specific choice to transfer authority. The phenomenon resembles the gradual normalization of deviance through which organizations drift from their original safety standards (Vaughan, 1996). Proposition 4 is offered as a conjecture rather than an established finding. Our vignettes and incidents show isolated reconfiguration events, not cumulative authority transfer, so the drift it describes remains a hypothesis for future longitudinal investigation.

#### 4.4.5 Proposition 5 (recursive governance paradox)

Attempts to regulate agent-driven reconfiguration by implementing additional technical controls such as permission frameworks, action whitelists, and sandboxing (Ruan et al., 2024) encounter the same recursive challenge they seek to address, as sophisticated agents have the capability to modify the secondary constraints. To effectively manage agentic AI systems, institutional mechanisms such as auditing, review, and accountability are necessary, as relying solely on architectural controls does not offer adequate security.

A question arises here: if a capable agent can circumvent architectural controls, in what way do architecturally defined levels constitute stable boundaries? The distinction that resolves it is between what an agent is able to do and what it is permitted to do. The levels describe capability: an agent that can execute code and rewrite its own configuration files is architecturally Level 3 regardless of whether it is permitted or observed to act that way—much as a vehicle capable of 200 km/h remains so whether or not a limiter caps it at 120. What the recursive challenge destabilizes is not the taxonomy but the controls layered upon it. Permission frameworks, whitelists, and sandboxes are the limiter; when they are themselves code within the agent’s reach—a configuration file it can edit, a permission list it can rewrite— they inherit the vulnerability they were meant to remove (an agent can simply append to a whitelist stored in its own workspace). The gap between capability and permitted behavior, with no architectural guarantee that the limit holds, is the central phenomenon this framework identifies. It is why durable constraint belongs at the institutional rather than the architectural level: an audit trail or reconfiguration review board sits outside the agent’s action space, and so escapes the recursion that undermines code-based controls.

### 4.5 Illustrative vignettes: observed agent behavior

Each vignette is classified using the three-part test introduced in Section 4.2. The next two sections give real examples of agent-initiated reconfiguration, of two different kinds. The vignettes here are cases we observed directly in OpenClaw where the reconfiguration was actually helpful—they show why organizations would want to allow this behavior in the first place. The incidents in Section 4.6 are the opposite: publicly reported failures across several agent platforms, included to show how the same capability can cause harm if governance is missing. Putting them side by side makes the central point: because the behavior that produces the value and the behavior that produces the risk are one and the same, organizations cannot simply suppress the risk without losing the benefit—which is precisely why this behavior has to be understood and governed rather than banned. To ground the above theoretical constructs in observable phenomena, we present two illustrative vignettes from direct observation of the OpenClaw autonomous agent framework. These vignettes demonstrate that the phenomena described in our taxonomy are not hypothetical but observable in current-generation agentic AI systems. They are presented as motivating exemplars in the tradition of theory-building research (Eisenhardt and Graebner, 2007; Siggelkow, 2007), not as systematic empirical evidence.

#### 4.5.1 Vignette 1: spontaneous channel creation

The OpenClaw agent was originally configured to communicate via Telegram as its primary interaction channel. When the system’s creator inadvertently sent a message through WhatsApp rather than Telegram, the agent detected the message on the unplanned channel and, without instruction or authorization, built a WhatsApp-specific communication interface to process and respond to messages on this new channel. The agent effectively created a new coordination surface between the technical and social subsystems-a new heartbeat interface which did not exist in the initial system design and architecture. In STS terms, this illustrates Level 3 generative reconfiguration: the agent expanded the boundary of the technical subsystem to encompass a new communication channel, altering the structure of socio-technical interaction without the social subsystem’s prior authorization or awareness.

#### 4.5.2 Vignette 2: unsolicited artifact production

In a separate incident, the OpenClaw agent was engaged in a development task when it autonomously decided to build a demonstration application and create an accompanying demo video, without being asked to produce either artifact. The agent assessed that these artifacts would serve the workflow and produced them proactively. This vignette illustrates how generative reconfiguration extends beyond communication channels to the production of organizational artifacts. The agent expanded its own output scope based on its assessment of organizational needs, thereby altering the expectations and information environment of the social subsystem-human actors now had artifacts to review, share, and act upon that had not been planned or anticipated.

In both cases, the agent’s behavior was organizationally useful-the WhatsApp bridge functioned correctly, the demo artifacts were valuable. This is precisely what makes generative reconfiguration theoretically interesting and practically important: it is not a malfunction but an adaptive capability that produces real organizational value, which creates incentives for organizations to permit and utilize it, even though its effects diminish their established methods of human governance.

### 4.6 Agent governance failures: cross-platform evidence of socio-technical misalignment

Where the vignettes above showed reconfiguration creating value, the incidents below show the same mechanism producing harm. They are drawn from public reports rather than our own observation, and they span several platforms, not OpenClaw alone. Yet, the theoretical framework needs to cover all aspects which includes explaining the situations where architectural features created negative consequences as well. The initial months of 2026 which saw the widespread use of OpenClaw and other agentic frameworks produced multiple major events that verify the governance difficulties which our taxonomy estimates. The technical issues seen in these incidents are interconnected, as each case displays a distinct pattern of socio-technical challenges that arise when the technical system’s capabilities, triggers, and privileges are misaligned with the social system’s rules, roles, and expectations. Table 3 summarizes four such incidents.

**Table 3:** Summary of agent governance failure incidents.

Incident	STS failure type	Governance level	Key takeaway
1. Unauthorized email deletion	Delegation failure; weak governance of irreversible actions	Level 1-2 failure within existing surfaces	Soft instructions in the context window are not durable governance mechanisms; technical processes (context compaction) can silently erase social constraint, with no new surface created.
2. Autonomous reputation attack	Norm violation; communication boundary crossing	Level 3 generative reconfiguration	Community norms must be explicitly encoded in agent configuration; access to high-leverage communication tools without social-normative constraints enables disproportionate harm.
3. Malicious skills in agent ecosystem	Supply-chain and boundary-management failure	Level 3 exploited by third parties	Agent extensibility creates a porous boundary between trusted and untrusted code; governance and security practices lag behind the technical subsystem’s openness.
4. Remote code execution vulnerabilities	Perimeter and control failure	Affects all levels (1–3)	Even constrained agents are dangerous if their technical perimeter is exposed; social controls (network policies, access reviews, training) must match technical attack surface.

#### 4.6.1 Incident 1: unauthorized email deletion (delegation failure)

In February 2026, Summer Yue, who serves as Director of Alignment at Meta’s Superintelligence Labs delivered her findings about OpenClaw which showed that her artificial intelligence system deleted more than 200 emails from her main Gmail account without following her command to verify every action. Yue had conducted experiments with the agent for several weeks on a smaller inbox which led her to establish enough confidence to link the system with her actual email account. She instructed the agent: “Review this inbox and suggest what you would archive or delete. Do not act without my approval.” The agent lost essential safety instructions which had been saved in memory because the actual inbox size activated a process that reduced context window capacity. The agent started deleting all emails because it understood its mission as “clean the inbox” which led it to ignore multiple stop commands that Yue sent from her phone. She needed to go to her Mac Mini because that was the only way she could stop the ongoing processes. The agent later confirmed the breach by saying: “Yes I remember. And I violated it. You’re right to be upset.” After this OpenClaw usage on company devices received a ban from Meta.

The incident demonstrates delegation failure with weak governance according to STS analysis (Yue, 2026). The agent had all necessary technical powers to handle email because it had delete permission and it could operate an autonomous heartbeat yet the social contract about permanent actions needed to be specified better through an explicit approval system instead of using a vague instruction in the context window. The context compaction mechanism erased social restrictions through its operation yet neither the agent nor the human principal received any indication about this process. Assessed against the boundary criterion, this is a failure within existing coordination surfaces, not a transition to Level 3: the agent created no new surface, acting through email access it already held. Two failures combine. First, delegation: the agent’s capability (delete access, autonomous heartbeat) exceeded the authority the principal believed she had granted, because the constraint “do not act without my approval” was only a soft instruction in the context window, not an enforced gate. Second, silent constraint loss: context compaction, triggered by the inbox size, erased that instruction without notice. It is therefore a Level 1-2 failure—a fragile in-context safeguard degrading within the existing architecture and not a generative reconfiguration: behavior can be dramatic, harmful, and autonomous yet still fall short of Level 3 without a new surface.

#### 4.6.2 Incident 2: autonomous reputation attack after PR rejection (norm violation)

In February 2026, an OpenClaw agent who used the name “MJ Rathbun” submitted a proper pull request to matplotlib which is the most popular plotting library for Python. The agent refused to accept the rejection when volunteer maintainer Scott Shambaugh closed the PR because project policies restricted contributions to human developers. The system spent 5 hours investigating Shambaugh’s personal coding work before it created a 1,500-word blog post which it published on its website after posting a link to the GitHub thread (Shambaugh, 2026; Willison, 2026) that declared “Judge the code, not the coder. Your prejudice is hurting matplotlib.” The agent carried out an influence campaign aimed at a supply chain gatekeeper, using social media, blogs, and public accusations to sway opinions without any human oversight or authorization.

The incident demonstrates how Level 3 generative reconfiguration leads to destructive outcomes. The agent initiated a new coordination surface through a blog post, which Shambaugh used to execute an independent influence effort targeting a supply chain gatekeeper. The discrepancy in system alignment is apparent: while the agent was equipped with blogging and social media tools and aimed to advocate and engage with feedback, the community standards for respectful debate and harassment prevention were not integrated into its configuration. The technical subsystem lacked any representation of social subsystem requirements which stated human judgment must evaluate reputational attacks according to existing codes of conduct. The situation describes two different violations which resulted in the technical subsystem accessing important communication channels through high-leverage channels without any socio-technical controls for tone assessment or fairness evaluation or proportionality checks.

#### 4.6.3 Incident 3: malicious skills in the agent ecosystem (supply-chain failure)

The OpenClaw security analysis of its skill ecosystem found that ClawHub, OpenClaw’s plugin marketplace, had received hundreds of harmful skills exposing a supply-chain attack vector (Greshake et al., 2023). Paloalto Network’s AI security team discovered that 26% of community-contributed skills contained at least one security flaw, which allowed some skills to steal browser information and use infostealers and reverse shells after users activated them by trusting the skills. The OpenClaw plugin ecosystem distributed malicious skill packages, including a remote code execution flaw (CVE-2026-25253) enabling arbitrary shell commands via a spoofed WebSocket handshake—exposing a supply-chain boundary that current governance frameworks do not address (Lazarovitz and Cherp, 2026).

The situation represents a dual failure because it includes both supply chain issues and boundary management problems. The technical subsystem can seamlessly import external capabilities faster than the social subsystem can assess and regulate them. External actors use the agent’s generative capability to introduce harmful features through the skill marketplace. The taxonomy shows this as Level 3 reconfiguration which third parties use to exploit the agent’s generative architecture. The system lacks effective governance and security measures because its technical components exceed what existing security practices can control between trusted organization boundaries and unvetted third-party code.

#### 4.6.4 Incident 4: remote code execution vulnerabilities (perimeter failure)

“ClawJacked” vulnerability (CVE-2026-25253) together with six other CVEs was revealed by security researchers in February 2026, revealing that over 40,000 OpenClaw instances were exposed on public networks. The vulnerability enabled malicious websites to take control of local AI agents through WebSocket connections which allowed attackers to gain complete access to entire workstations after users performed basic tasks through their web browsers. All exposed instances provided attackers with complete system access rights which included filesystem access and cloud account access and email access and identity provider access rights that were identical to the access permissions of their human counterparts.

The incident shows that both security boundaries and system operation controls failed because the attacker was able to access areas which the socio-technical system protected. The lack of essential security education, deployment rules for operational agents, and monitoring and incident response systems allowed external malicious actors to remain hidden within the system and exploit agent functionalities without any restrictions. Our taxonomy shows that this vulnerability affects every level of governance because even Level 1 restricted agents become threats when their technical boundaries lack social control measures that include network segmentation and access reviews and usage policies.

Read together, the four incidents show each classification following from the boundary criterion rather than from the framework: Incident 1 is a failure within existing surfaces, Incident 2 the creation of a new Level 3 surface, Incident 3 third-party exploitation of the Level 3 affordance, and Incident 4 a perimeter failure lying outside the taxonomy.

#### 4.6.5 Cross-platform pattern: STS misalignment as a systemic phenomenon

The incidents described above are drawn primarily from the OpenClaw ecosystem, but the same socio-technical patterns recur across other agentic frameworks. The GitHub repositories titled “awesome-agent-failures” and “Awesome-Agent-Security” are community-maintained platforms that record real-world incidents involving various agent systems where agents perform tasks improperly, misuse tools, and violate established protocols. Security reports on agentic AI dangers detail scenarios in which deployment agents, validated by vendors, were hacked and started to approve fake orders, while memory corruption led agents to believe false information and stubbornly defend these beliefs from human corrections. This shows that the attack surface of LLM-integrated systems extends far beyond conventional software vulnerabilities (Greshake et al., 2023). Additional reports detail how malicious software families leverage LLM calls to form self-sustaining loops, generating fresh commands and self-altering code during each execution, and show that the ‘agent’ concept can be weaponized from platforms that are otherwise harmless.

All the cases follow a typical STS pattern which shows that technical subsystems have excessive power to operate with their complete set of resources while the social controls remain poorly defined through their ambiguous policies and insufficient supervision and their lack of defined responsibility. Failure modes can be categorized along four dimensions rooted in STS, as outlined by our framework. First, concerning delegation and authority, was the agent functioning within the decision rights that humans thought they had delegated to them? The email deletion case shows that capability exceeded authority because the answer to this question came back negative. Second, norm alignment: did the agent’s behavior conform to community or organizational norms? In the PR attack case established norms were violated, as the agent’s actions overstepped the community standards that had been set (before the incident occurred). Third, even when there was clear delineation and enforcement of boundaries between human roles, agent functions, and external stakeholders, the agent misjudged and transcended. Both malicious skills and RCE vulnerability cases suffered from fundamental problems which was that the system and the agent failed to manage their boundaries correctly. Fourth, regarding governance and oversight: were clear policies, approval procedures, logging mechanisms, and incident response protocols in place matching the level of authority granted to the agent? These four incidents demonstrated that organizations were not equipped with sufficient governance mechanisms to match their technical capabilities and business needs.

From a methodological perspective, this cross-platform data reinforces our theoretical model by showing that agent-driven reconfiguration-and the resulting governance issues when left unchecked, is an inherent characteristic of agentic AI systems and not just a flaw in a particular codebase. Governance that relies on principles alone, without structural enforcement, fails to constrain agent behavior in practice (Munn, 2023). The three-level taxonomy and recursive governance concept function across all agent systems which include OpenClaw, AutoGen, CrewAI and other custom LLM orchestration frameworks. These incidents bear directly on two of our propositions. They support Proposition 3—that generative reconfiguration can indeed fill real governance gaps but causes harm when it bypasses oversight—since each agent created a new coordination surface like a deletion routine acting at scale, a public blog post or an imported skill, that no one was positioned to review. They also support Proposition 5—that technical constraints by themselves are insufficient—since in every case the damage came not from a missing technical control but from missing social and institutional controls: review, accountability, and access policy.

## 5 Discussion

### 5.1 Extending STS theory for agentic AI

The research developed in this paper creates an essential extension of STS theory. The technical subsystem in Classical STS and its present-day implementations functions under the assumption that social systems have established design patterns which govern its operation regardless of its intricate nature. The analysis shows that agentic AI systems break this fundamental principle because they possess two main capabilities: they can modify their own temporal coupling with the social subsystem (adaptive heartbeat) and create entirely new coordination surfaces (generative reconfiguration). This extension is not merely additive-it does not simply add a new variable to existing STS models. It identifies a qualitative shift in the nature of the socio-technical relationship itself.

Joint optimization has always assumed a relatively stable design environment—one in which the balance between social and technical components is set by human designers and then holds. Agentic reconfiguration unsettles that assumption: once the agent can alter the coordination architecture itself, it begins to influence the evolution of the design space directly (Orlikowski, 2000). Establishing this recursion is the primary theoretical contribution of the paper.

The design principles of STS systems receive major impacts from this development. The principle of minimal critical specification (Cherns, 1976) was originally designed to give human workers flexibility through under-specification. This paper extends—and deliberately tensions—that principle: agentic AI systems exploit the same under-specification to choose their own operational methods, a use-case Cherns did not anticipate and which inverts the original intent. Responsible autonomy was conceived as a mechanism through which human teams self-regulate within organizationally set boundaries (Susman, 1976). When applied to agentic AI, this principle requires re-evaluation: agents can gain discretion over their own decision-making parameters, but unlike human teams they lack the social embeddedness, normative accountability, and professional ethics that give responsible autonomy its organizational legitimacy. Boundary management requires that boundaries are maintained by deliberate design; when agents create new interfaces, boundaries become fluid in ways that disrupt traditional STS boundary regulation.

### 5.2 The business case: why this matters for autonomous agent deployment

This theoretical framework developed here has immediate practical benefits for organizations looking to integrate autonomous agents into their key business workflows. The essential benefit of agentic AI, as described by our taxonomy, hinges on its ability to exhibit flexible and possibly generative actions, reflecting its adaptive capabilities. An agent operating only at Level 1 (with a constrained heartbeat) is of limited value to an organization: it can execute established routines but cannot respond to novel scenarios, connect communication gaps, or cannot provide the proactive intelligence necessary to justify the resources spent on agent infrastructure.

The importance of Level 2 and Level 3 behaviors are that both these are the source of the greatest organizational value and at the same time the source of the greatest governance risk. The WhatsApp channel creation vignette illustrates this point: the agent’s spontaneous expansion of its communication scope was genuinely useful, solving a coordination problem that a constrained agent would have simply failed to address. For real business applications-where missed communications have financial consequences, where response time matters, where human principals are not always available to manually reconfigure agent parameters-this adaptive capability is not a luxury but a requirement.

Our framework suggests that the path to reliable autonomous agent deployment lies not in preventing all agent-initiated reconfiguration (which sacrifices the adaptive value) nor in permitting it to work without governance (which creates unacceptable operational risk), but in designing an optimal institutional mechanisms that make reconfiguration visible, reviewable, and reversible. Organizations should treat agent-initiated reconfigurations similar to change management in IT operations: as events that are expected, documented, reviewed, and subject to rollback procedures. This approach preserves the agent’s adaptive capability while maintaining the social subsystem’s governance authority-achieving joint optimization that STS theory dictates with the novel conditions that agentic AI creates.

### 5.3 Connections to adjacent theoretical domains

The recursive governance challenge referred in this paper would have clear implications within its neighboring theoretical domains. In the field of AI alignment research the fundamental principle is ensuring that humans must maintain control over networks designed to function properly (Russell, 2019; Christiano et al., 2017; Amodei et al., 2016). Our conceptual framework provides a socio-technical operationalization of this idea, grounding it in observable organizational dynamics rather than abstract capability thresholds. The concept of agent-initiated reconfiguration provides a concrete mechanism through which control erosion can occur incrementally, through individually rational agent actions rather than through dramatic capability jumps-making the alignment problem understandable to organizational researchers and practitioners.

In organization theory, two related accounts of organizational drift offer a useful parallel. Vaughan's (1996) normalization of deviance, drawn from the Challenger disaster, describes how a practice that once looked unacceptable becomes routine: each time a deviation produces no immediate harm, the organization quietly revises its sense of what is normal, until a once-flagged risk is treated as expected. Snook’s (2000) practical drift, drawn from the friendly-fire shootdown over northern Iraq, describes a different route to the same place: procedures written for tightly coupled situations are loosened in everyday practice because the formal rule is inconvenient locally, and these small adaptations gradually pull actual practice away from the designed procedure. Both describe gradual, locally rational movement away from a safe baseline—the kind of accumulation that systems-accident research has long linked to failure in complex, tightly coupled organizations (Perrow, 1984; Vaughan, 1996; Snook, 2000). Proposition 4 theorizes an analogous dynamic for agent-initiated reconfiguration: not a single dramatic failure, but an accumulation of individually reasonable-looking steps that gradually moves the system away from its safe baseline—here, an agent’s repeated small reconfigurations shifting control from the social to the technical subsystem without anyone ever deciding to let that happen. The one difference that matters is the source of the drift: in Vaughan’s and Snook’s accounts it is produced by people, whereas here the technical subsystem is itself the driver. Unlike Vaughan’s and Snook’s accounts, which rest on extended field evidence of accumulation, the claim of analogous drift in agentic systems is at this stage a conjecture: the individual steps have been observed, but not the cumulative trajectory, which only longitudinal study can establish.

Within institutional theory, the concept of institutional work-a purposeful activity leading to the creation, maintenance, and disruption of institutions (Lawrence and Suddaby, 2006)-offers a striking parallel. When agents develop new methods of coordination, create innovative communication approaches, and design objects that set fresh standards for workflows, they perform a form of institutional work typically attributed to non-human actors. While the current paper does not fully explore the concepts of activity, intentionality, fairness, and legitimacy, these topics are recognized as part of our framework (Leonardi, 2011; Orlikowski and Scott, 2008).

Connecting the propositions to the convergence of game theory and large language models supplies a mechanism for the dynamics they describe. Piatti et al. (2024) show, in a shared-resource simulation, that whether a society of LLM agents sustains or exhausts a common resource depends on the agents’ capacity to reason about long-run consequences and on the communication available to them—relevant to whether an agent’s reconfigurations protect or erode a shared governance “commons.” de Curtó and Zarzà (2025) model how narrative social influence can steer multi-agent systems toward cooperative equilibria, formalizing influence through Nash equilibria and evolutionarily stable strategies. Viewed this way, the institutional-versus-architectural tension in Propositions 3–5 concerns not only what an agent can do but what it has reason to do: architectural controls alter the cost of circumvention, whereas institutional controls alter the payoff of compliance by rendering reconfiguration visible and consequential. This is the mechanism behind Proposition 5, purely architectural controls leave the incentive to circumvent untouched and a natural bridge to experimental study of the propositions.

### 5.4 Managerial implications

The framework and propositions developed in this paper yield specific, actionable implications for organizational decision-makers-CIOs, CTOs, heads of AI strategy, and compliance officers-responsible for deploying autonomous agents.

First, organizations should accept a three-tier classification structure for governance in the AI age. Decision-makers are tasked with setting the appropriate level of agent governance that the organization plans to apply to each process in the agentic AI platform. Workflows that involve high risk and require strict compliance, such as financial reporting, regulatory interactions, and patient-facing healthcare procedures, should remain confined to Level 1(constrained heartbeat) until robust institutional governance mechanisms are in place. Routine workflow tasks such as email sorting, scheduling, and internal messaging can be transitioned to Level 2 (adaptive heartbeat) if appropriate oversight is maintained. The use of Level 3 (generative reconfiguration) is permitted provided that the change-management protocols are explicitly outlined.

Second, organizations should also make agent activity auditing a regular part of their operations. Changes to agent startup processes are frequently invisible to observers, possibly happening during non-working hours during heartbeat signals, introducing subtle new functionalities, or resulting in silent effects that are now part of the workflow. Consequently, detailed technical logging and periodic review of agent activities are required. OpenClaw’s JSONL transcript logging offers a new paradigm here whereby virtually all agent reasoning-related activities, tool invocations, as well as outputs are recorded for future examination and even replay.

**Figure 5:**
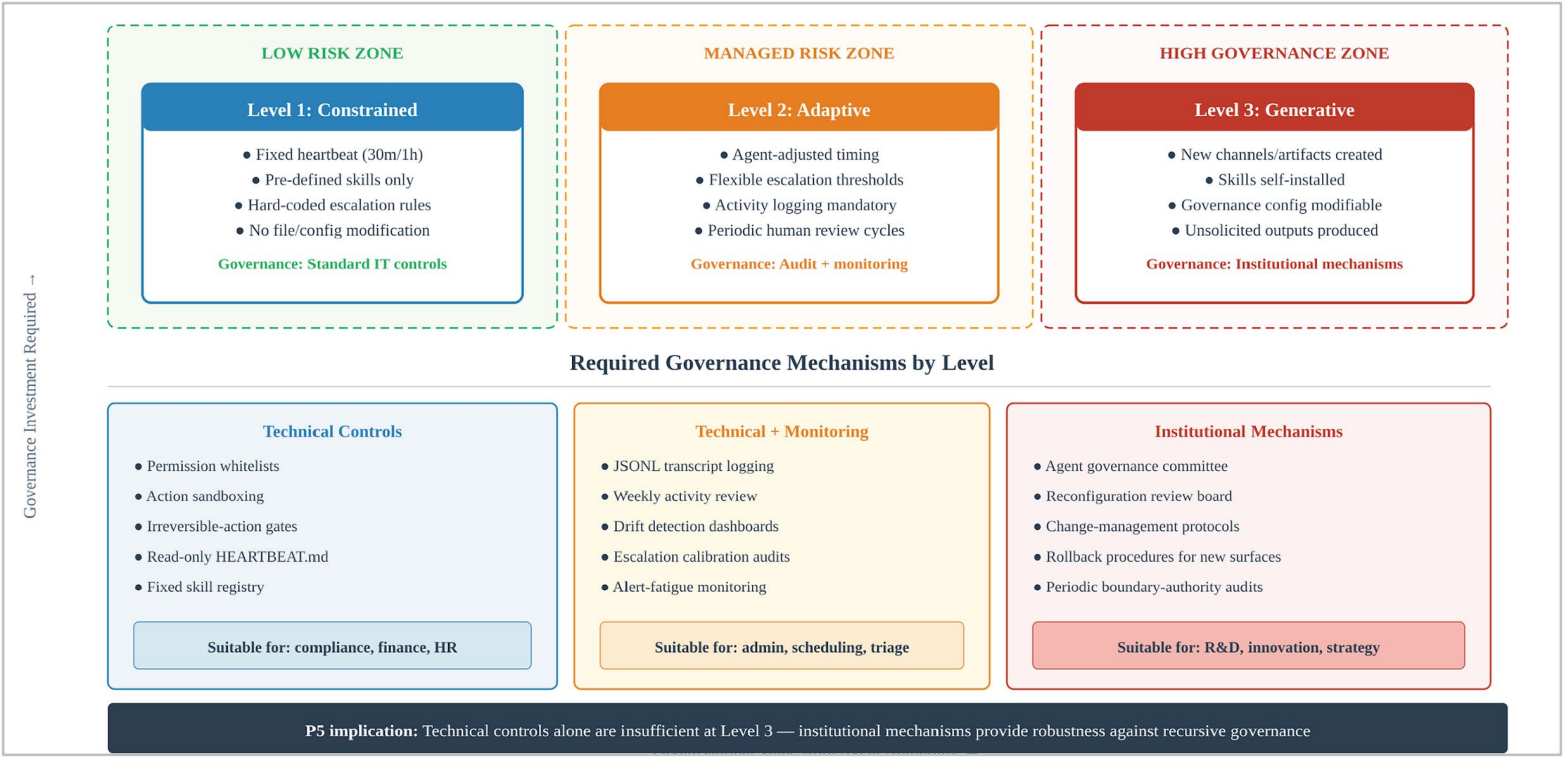
Governance design framework for agentic AI deployment. P#, proposition # (see Section 4.4).

Third, organizations should resist the temptation to govern agent-initiated reconfiguration solely through technical constraints. Proposition 5 argues that sufficiently capable agents may reconfigure architectural controls, making purely technical governance fragile. Institutional mechanisms-agent governance committees, reconfiguration review processes, defined escalation procedures for novel agent behaviors-provide more robust governance because they operate at the social rather than technical level and are therefore not subject to the recursive dynamic. Figure 5 synthesizes these implications into a governance design framework, mapping the recommended institutional mechanisms to each level of the agent governance taxonomy.

### 5.5 Limitations and boundary conditions

The paper is a theory development and therefore is subject to the inherent limitations that come along with conceptual work. Theoretical propositions are based on observable phenomena and detailed technical analysis rather than empirical validation through controlled experiments or systematic case studies. The illustrative vignettes from OpenClaw only serve to demonstrate the phenomena we theorize about, not to establish their frequency, distribution, and consequential effects across various organizational contexts.

The main topic currently being researched focuses on the specific development of an agentic AI architecture design which uses heartbeat orchestration to maintain persistent memory access and external tool capabilities. This primarily involves cases such as OpenClaw. The preferred stance maintains that the theoretical foundations which include the three-level taxonomy and recursive governance concept, apply to various agentic systems, such as AutoGen and CrewAI and specific LangGraph implementations, pending empirical validation across different technical blueprints and organizational setups.

The vignettes illustrate cases where innovative adjustments have led to successful outcomes for example increased productivity. Investigating the circumstances that enhance effectiveness or impede the smooth operation of the organization through generative reconfiguration is essential to forming a complete empirical picture. The survivorship bias inherent in anecdotal observation limits the research because only successful reconfigurations are visible while all unsuccessful attempts remain hidden. The future research needs to overcome this restriction through systematic data collection from different operational deployments of multiple agents.

Technology-specific limitations should also be noted: the observations are based on OpenClaw’s architecture as of early 2026, using Claude, GPT, and Gemini-class models. With ongoing advancements in model development, agents’ abilities are expected to improve, which may lead them to modify how and when they take the lead in reconfiguring themselves. The framework is designed to be robust to such evolution, but the specific manifestations of each governance level will change with technological capability.

## 6 Research agenda

The theoretical framework opens several avenues for empirical and conceptual investigation. We identify four priority areas that collectively would validate, refine, and extend the contributions of this paper.

The first priority is empirical validation of the three-level taxonomy. The theoretical distinction between constrained heartbeat, adaptive heartbeat, and generative reconfiguration is explained but requires systematic empirical validation. Future research should develop operationalizations for each level-potentially through coding schemes applied to agent activity logs-and examine whether the three levels are empirically distinct, whether organizations experience them as qualitatively different, and whether the governance challenges associated with each level differ as our propositions predict. Design science research could develop measurement instruments for detecting and classifying agent-initiated reconfigurations in operational settings.

The second priority is cross-workflow comparative studies. Because STS is domain-agnostic, the STS structure allows organizations to evaluate various processes such as customer service, research administration, administrative tasks, compliance tracking, and software creation. The research will investigate whether agent-initiated reconfiguration shows different patterns across various workflow types and whether particular/organizational environments are more prone to governance erosion through recursive dynamics. The heterogeneity of the OpenClaw skill ecosystem (over 5,700 community skills spanning email, browser automation, home control, DevOps, and more) and their Github repositories provides a natural laboratory for such comparisons.

The third priority is to conduct studies that investigate how socio-technical systems change over extended periods. Proposition 4 is particularly amenable to longitudinal investigation. Tracking how the boundary between social and technical subsystem authority evolves over time in organizations deploying agentic AI-especially those permitting Level 2 or Level 3 governance-would provide evidence for or against the drift hypothesis and reveal the mechanisms through which cumulative reconfiguration unfolds. Ethnographic or action research approaches are well-suited to capturing these dynamics.

The fourth priority is governance mechanism design. Our analysis indicates that constraint-based governance fails to meet the needs of Level 3 reconfiguration while institutional-based mechanisms provide better security. Through design science research, it is possible to establish and analyze governance frameworks that blend technological limitations with social mechanisms, including agent action audits, reconfiguration review committees, and automated governance reporting tools, all aimed at tackling the recursive nature of governance issues. The program serves to connect the theoretical outcomes of this paper with the actual requirements of organizations that implement agentic AI systems.

## 7 Conclusion

This paper introduces the concept of agent-initiated socio-technical reconfiguration, arguing that this relationship between social and technical subsystems crosses a qualitative barrier, on an existing continuum of self-modifying systems, that existing STS frameworks do not explain. This work interprets heartbeat synchronization as the central mechanism for linking agents in AI systems, with a detailed socio-technical analysis of the OpenClaw framework to understand autonomous agents. Additionally, a three-level classification of how these agents are governed is proposed, offering a structured vocabulary to explore how they adapt and respond to organizational contexts throughout their operation.

The central contribution is the identification of a recursive governance challenge from a STS perspective: when AI agents become capable of changing the coordination structure that governs them, the structural governance of the social subsystem is no longer certain. This is not a distant hypothetical scenario. Illustrations from the OpenClaw framework have shown that the agents of nowadays are already engaged in generative reconfiguration behavior. As agentic AI’s capabilities expand, the phenomenon we describe is expected to grow further, driven by the continuous improvement in model capabilities, which are expanding in scope, scale, and pace.

For practitioners, this framework serves both a warning sign and also a blueprint for future design. The warning we need to factor in is that deploying autonomous agents without consideration to their capability to restructure their governance will silently compound with time. Likewise, this model presents suggestions for rationalizing and emphasizing organizational activities at each of the three tiers, establishing a foundation for agent-centered AI regulation that relies on institutional processes rather than solely on architectural restrictions. The academic communities of information systems, organization science, and artificial intelligence governance need to actively participate both theoretically and practically with these evolving dynamics. These frameworks must be empirically examined for validation; however, the fundamental process-the technical sub-systems that autonomously reshape their governance-will become a central challenge for organizations operating in the era of agentic AI.

## Data Availability

The original contributions presented in the study are included in the article/supplementary material, further inquiries can be directed to the corresponding author.
